# Holistic OR domain modeling: a semantic scene graph approach

**DOI:** 10.1007/s11548-023-03022-w

**Published:** 2023-10-12

**Authors:** Ege Özsoy, Tobias Czempiel, Evin Pınar Örnek, Ulrich Eck, Federico Tombari, Nassir Navab

**Affiliations:** 1 Computer Aided Medical Procedures, Technische Universität München, Garching, Germany; 2 Google, Zurich, Switzerland

**Keywords:** Semantic scene graph, Surgical scene understanding, 3D, 4D-OR

## Abstract

**Purpose:**

Surgical procedures take place in highly complex operating rooms (OR), involving medical staff, patients, devices and their interactions. Until now, only medical professionals are capable of comprehending these intricate links and interactions. This work advances the field toward automated, comprehensive and semantic understanding and modeling of the OR domain by introducing semantic scene graphs (SSG) as a novel approach to describing and summarizing surgical environments in a structured and semantically rich manner.

**Methods:**

We create the first open-source 4D SSG dataset. 4D-OR includes simulated total knee replacement surgeries captured by RGB-D sensors in a realistic OR simulation center. It includes annotations for SSGs, human and object pose, clinical roles and surgical phase labels. We introduce a neural network-based SSG generation pipeline for semantic reasoning in the OR and apply our approach to two downstream tasks: clinical role prediction and surgical phase recognition.

**Results:**

We show that our pipeline can successfully reason within the OR domain. The capabilities of our scene graphs are further highlighted by their successful application to clinical role prediction and surgical phase recognition tasks.

**Conclusion:**

This work paves the way for multimodal holistic operating room modeling, with the potential to significantly enhance the state of the art in surgical data analysis, such as enabling more efficient and precise decision-making during surgical procedures, and ultimately improving patient safety and surgical outcomes. We release our code and dataset at github.com/egeozsoy/4D-OR.

## Introduction

**Fig. 1:**
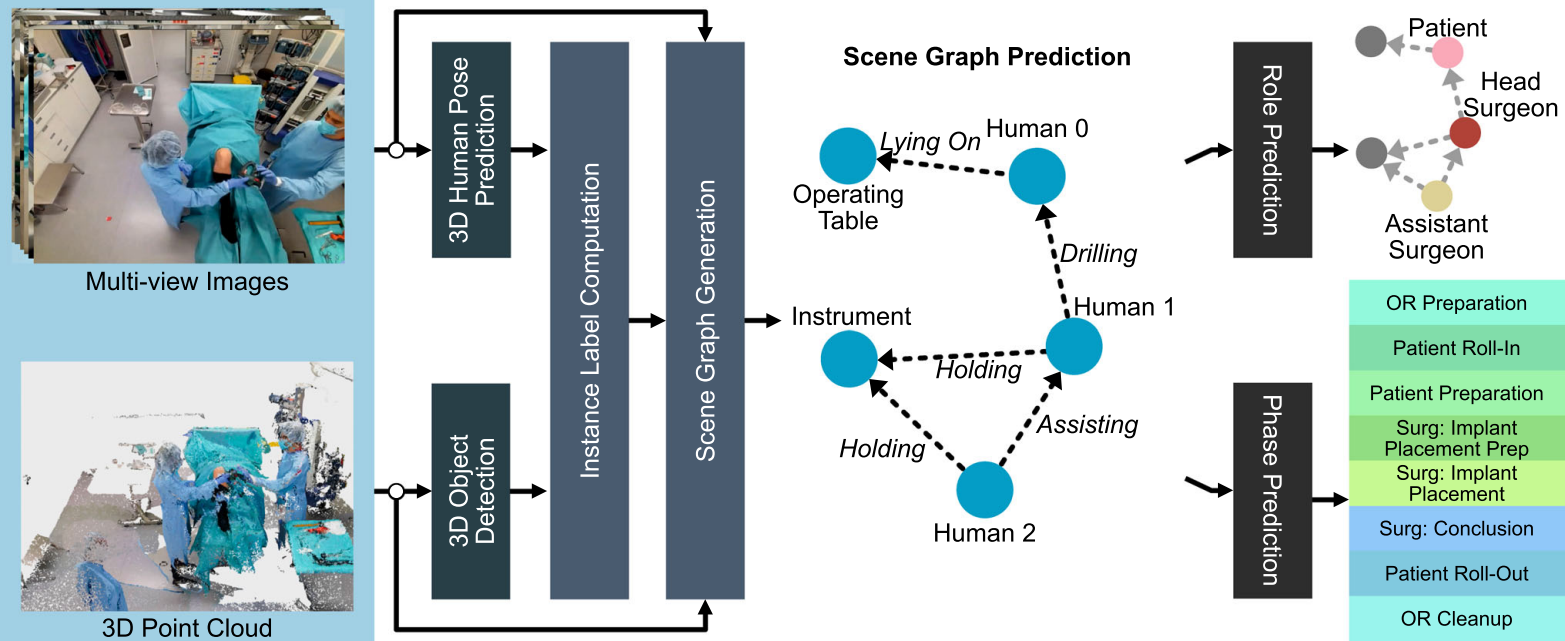
An overview of our scene graph generation pipeline. We predict 3D human poses from images and object bounding boxes from point clouds and assign an instance label to every point. The scene graph generation then uses the fused point cloud, instance labels and images to predict the relations between the nodes, resulting in a semantically rich graphical representation

Holistic and automated understanding of the OR is a crucial step toward the next generation of computer-assisted interventions [1–4]. The nature of the ORs, which are highly complex and variable, with numerous medical staff, patient and medical equipment, and their diverse interactions make semantic reasoning in the OR and about surgical procedures fundamentally challenging.

So far, the surgical data science (SDS) community has primarily focused on analyzing specific tasks, such as surgical phase recognition, instrument recognition, human pose estimation, hand tracking and situational awareness estimation [5–12]. For a more complete understanding of the procedures, it is essential to establish models that can accurately untangle the numerous participants, objects and their interactions, considering the OR as one interwoven entity instead of several separate activities. This would allow digital systems, like medical robots, imaging equipment or user interfaces, to act autonomously according to the needs of the surgery, resulting in an optimized workspace and improving patient outcomes.

Scene graphs are used to abstract image information by representing objects or individuals as nodes, and relationships between nodes as edges [13]. This powerful symbolic representation have shown benefits in a wide range of applications, such as image generation [14], scene manipulation [15], action recognition [16] and 3D camera relocalization [17]. While there have been many successful applications of scene understanding methods in computer vision on benchmark datasets for everyday tasks [18–22], the content and complexity of these datasets are generally simpler compared to a modern dynamic OR. Despite the potential for diverse applications, scene graphs have not yet been employed to model the unique 3D dynamics and complex semantic interactions that occur among various entities within an OR setting.

To facilitate the training and evaluation of an OR-specific scene graph generation model, a 4D external view OR dataset, with SSG annotations and downstream task annotations, is needed. Sharghi et al. [23] capture different robot-assisted interventions, focusing on phase recognition lacking more semantic annotations. Srivastav et al. [10] introduced the only publicly available external view OR dataset, with synchronized multiview frames and human pose annotations. This dataset significantly contributes to advancing human pose recognition, but does not contain the semantic labels that would facilitate a more comprehensive modeling of the surgical scene. Additionally, the dataset is limited to single time points, omitting any 4D temporal information.

To this end, we introduce a new 4D operating room dataset, 4D-OR, which consists of 10 simulated knee surgeries annotated with human and object poses, semantic scene graphs, clinical roles and surgical phases. In conjunction with this dataset, we propose a novel, end-to-end, neural network-based method to generate SSGs for semantic reasoning in the OR. Given a scene, our network predicts a semantic scene graph that is structured, generalizable and lightweight, summarizing the entire scene with humans, objects and their complex interactions. Finally, we highlight the power of our semantic scene graph representation on clinical role prediction and surgical phase recognition tasks. In this extended work, building upon our previous work presented at MICCAI 2022 [24], we introduce an additional downstream task: *surgical phase recognition*. Furthermore, we refine our training approach to improve scene graph generation performance and offer a more comprehensive explanation of our dataset, its annotations and the underlying methodology (Fig. 1).

**Fig. 2:**
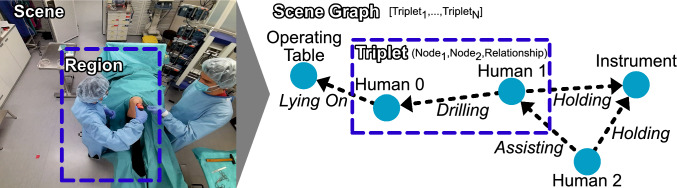
Region and scene with corresponding triplet and scene graph representation

## Methods

In this section, we delineate the methods employed in our study, focusing on the construction of semantic scene graphs, the development of our 4D-OR dataset, the implementation of our scene graph generation pipeline and the downstream tasks of clinical role prediction and surgical phase recognition.

### Semantic scene graphs

Semantic scene graphs (SSG) provide a structured representation of objects and their semantic relationships within an environment. They are defined by a set of tuples $\mathcal {G} = (\mathcal {N}, \mathcal {E})$, with $\mathcal {N} = \{n_{1},\ldots ,n_{n}\}$ a set of nodes and $\mathcal {E} \subseteq \mathcal {N} \times \mathcal {R} \times \mathcal {N}$ a set of directed edges with relationships $\mathcal {R} = \{r_{1},\ldots ,r_{M}\}$ [13]. Within a 3D scene, the corresponding SSG captures the entire environment including the location of each node. In the specific case of an OR, nodes in the graph encompass medical staff and equipment, such as the *anesthesia machine* or *operating table*. The edges represent the semantic interactions between nodes, such as a human *drilling* (into the bone of) the patient, as visualized in Fig. 2.

**Fig. 3:**
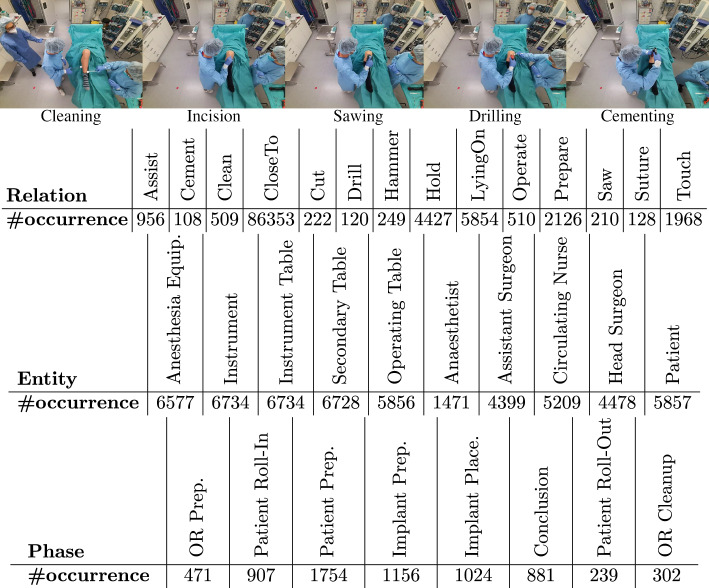
We visualize five exemplary relations, as well the number of occurrences of all relations, entities and surgical phases in the 4D-OR dataset

### 4D-OR dataset

To facilitate the modeling of intricate interactions in an OR using SSGs, we introduce the novel 4D-OR dataset. 4D-OR consists of ten simulated total knee replacement surgeries, which were conducted at a medical simulation center with input from orthopedic surgeons, ensuring a reasonable simulation of the surgical workflow. The actors, comprising three males and two females, were biomedical engineers doing their PhD and were informed by surgeons on the surgical procedure they were simulating. We chose total knee replacement as our intervention type, which is a representative orthopedic surgery, as it encompasses various steps and diverse interactions. 4D-OR contains a total of 6734 scenes, captured by six calibrated RGB-D Kinect sensors^1^ located at the OR ceiling. We empirically fixed the number of cameras to six to ensure a good trade-off between obtaining comprehensive OR coverage and ensuring practicality in hardware setup. The recording is done in one frame per second and is hardware synchronized across cameras. The average recording duration is 11 min, and the workflow can be seen as a simplified version of the real surgery. The roles of actors were switched regularly to create variety in the dataset. Some examples of activities present in the dataset can be seen in Fig. 3. Notably, 4D-OR is the only semantically annotated OR dataset. In addition to the images and fused 3D point cloud sequences, our dataset contains automatically annotated 6D human poses and 3D bounding boxes for medical equipment. Additionally, we annotate SSG for every time point, accompanied by the clinical roles of all humans present in the scene and surgical phases. For every frame, the authors created one annotation, in collaboration with medical experts.

### Scene graph generation

In the task of scene graph generation, the goal is to determine the objects and their semantic connections provided a visual input such as an image or point clouds. To this end, we present a novel end-to-end scene graph generation (SGG) pipeline, which is illustrated in Fig. 1. In our approach, we first identify humans and objects in the OR and extract their visual features. Then, we construct a semantic scene graph by predicting their pairwise relationships. We utilize state-of-the-art human and object pose estimation methods, VoxelPose [25] and Group-Free [26], to estimate the human and object poses, respectively. We design an instance label computation method that uses the predicted poses to assign each point in the point cloud an instance label. Furthermore, to ensure the detection of small and transparent medical instruments, which can be hard to localize in the point cloud, yet that are still represented in our scene graph, we introduce a virtual node termed *instrument* to represent interactions between humans and medical instruments. For predicting the pairwise relationships, we build upon 3DSSG [17].

3DSSG employs a neural network-based strategy to predict node relationships. It takes a point cloud and corresponding instance labels as input. Two PointNet [27]-based neural networks are utilized to calculate latent features. ObjPointNet processes the point clouds extracted at the object level. RelPointNet, on the other hand, processes object pairs, where for each object pair, it takes the union of the point clouds of the two objects as input. A graph convolutional network is then applied to contextualize the features of nodes and edges. Lastly, multilayer perceptrons are used to process the updated representations and predict object and relation classes. We train our scene graph generation network end-to-end, using the cross-entropy loss. For our SGG method, we design the following OR-specific modifications to 3DSSG:

**Multimodality by incorporating images:** The OR comprises numerous objects of varying sizes. Small, reflective or transparent instruments, such as *scissors* or *lancets*, are not always adequately captured by point clouds, even though their correct identification is crucial for many relationships. The vanilla 3DSSG often struggles with those relationships. Instead, we incorporate images alongside point clouds into our pipeline by extracting global image features using EfficientNet-B5 [28] and aggregating them with the PointNet features, enabling the usage of multimodal input for the scene graph generation.

**Data augmentation:** To simulate variations in the real world such as different clothing shades, lighting or object sizes, we augment the point clouds during training by applying random scale, position, orientation, brightness and hue changes. For point clouds associated with relationships, we augment the points of both objects separately, simulating them being in varying sizes or positions relative to each other. Finally, we employ a crop-to-hand augmentation, where we randomly crop the point cloud to the vicinity of the hands. This approach implicitly trains the network to concentrate on medical instruments when learning the relations such as *cutting, drilling* or *sawing*.

### Downstream tasks

We demonstrate the capabilities of our semantic scene graphs in two different downstream tasks: clinical role prediction and surgical phase recognition. The first aims to predict the role of medical staff in the OR, while the latter aims to determine the current phase of the surgery. Both tasks only utilize the SSG and no additional visual input. They benefit from the rich structural information provided by the SSG.

**Clinical role prediction:** To identify each individual’s role in the surgical setting, we first calculate a track *T* for each person using a Hungarian matching algorithm that leverages detected poses at each time stamp. Each track *T*, with a duration of *K*, consists of a selection of generated scene graphs $G_{Ti}$ where $i = {1,\ldots ,K}$ and a related human node $n_{Ti}$ for the track. The process of assigning clinical roles involves two primary steps: computing role likelihoods and assigning unique roles. For each track *T*, we compute a probability score indicating the likelihood of a specific role. We employ Graphormer [29], to process all the scene graphs within the track $G_T$. By designating nodes $n_{Ti}$ as *target* in the respective graph $G_{Ti}$, the network discerns which node embedding corresponds to the role. We compute the mean *target* node embedding over all the scene graphs in $G_T$ and predict clinical role scores using a linear layer trained with cross-entropy loss. Additionally, we introduce a heuristic-based method as a non-learning alternative for comparison, which uses the frequency of relations associated with each human node. For instance, the score for the *head surgeon* role increases with each *sawing* relation, while the score for the *patient* role increases with each *lying on* relation. Once clinical role likelihoods are computed, we deduce the clinical role of a human node by solving a matching problem. By retrieving role probabilities for each track, we match roles to nodes bijectively based on their probabilities, ensuring that each human node in the scene receives a distinct role, with the following algorithm:

1. For each human node, retrieve the associated role probabilities.
2. Identify the node with the highest probability for a specific role.
3. Assign that role to the node with the highest probability.
4. Remove the assigned role from the role probabilities of all other nodes.
5. Renormalize the role probabilities for the remaining nodes.
6. Repeat steps 2–5 until each node has a unique role assignment.

**Surgical phase recognition:** To detect the different phases of the surgical procedure, we first divide the surgery into eight distinct phases as enlisted in Table 3. For defining the phases, we follow the definitions of Sharghi et al. [23]. The phases with a “Surgery:” prefix imply main surgical operations, i.e., when the patient would be under anesthesia. Given the predicted scene graphs *G* from a surgery, we first enhance them by predicting the clinical roles of the medical staff. Then, we determine the correct phase corresponding to each scene by querying the scene graphs for specific triplets, such as “head surgeon sawing patient,” which we map to certain surgical phases. As our surgical phase recognition algorithm itself does not rely on a learning-based approach, it is transparent and does not need any additional annotations. As our semantic scene graphs already summarize the surgery at a high level, the detection of phases can be achieved with the following heuristics:

1. OR Preparation: SG does not include $\textbf{patient}$ and surgery did not start
2. Patient Roll-In: SG includes $\textbf{patient}$ and $operating\ \mathbf {operating\ table}$
3. Patient Preparation: SG includes $\mathbf {head\ surgeon}\ preparing\ \textbf{patient}$ and $\mathbf {assistant\ surgeon}\ preparing\ \textbf{patient}$
4. Implant Placement Preparation: SG includes $\mathbf {head\ surgeon}\ cutting\ \textbf{patient}$
5. Implant Placement: SG includes $\mathbf {head\ surgeon}\ hammering\ \textbf{patient}$
6. Conclusion: SG includes $\mathbf {head\ surgeon}\ cementing\ \textbf{patient}$
7. Patient Roll-Out: SG includes $\textbf{patient}$ and $operating\ \mathbf {operating\ table}$ and surgery is finished
8. OR Cleanup: SG does not include $\textbf{patient}$ and surgery is finished

**Fig. 4:**
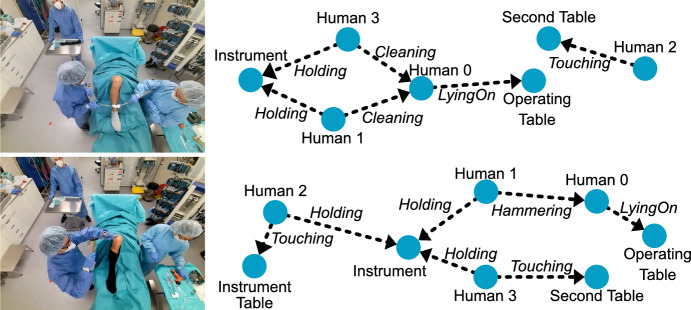
SGG results on two sample scenes. Only one input view is visualized for clarity

## Experimental setup

In this section, we present our experimental setup to evaluate our SSG pipeline and its application to clinical role prediction and surgical phase recognition.

**Implementation details**: The 4D-OR dataset is partitioned into training, validation and testing subsets, containing six, two and two takes, respectively. We adapt VoxelPose [25] to recognize 14 joints and train it for 20 epochs using a patient-pose weighted loss. Group-Free [26] is trained for 180 epochs. For SGG, we employ PointNet++ [30] as our feature extraction method, with a class balancing loss to address the challenge of rare relations. The learning rate is $3e-5$, and the network is trained on 4000 and 8000 points for predicting objects and relations, respectively. In this extended journal submission, we train our scene graph generation network for twice as many epochs as before, leading to improved results. Our pipeline, implemented in PyTorch and executed on a single GPU, attains an inference runtime of 2.2 FPS.

**Evaluation metrics**: To assess the performance of our proposed method, we use a set of comprehensive evaluation metrics. For human pose estimation, we utilize the Percentage of Correct Parts (PCP3D) metric. Object pose estimation is evaluated using average precision (AP) at a specified intersection over union (IoU) threshold. Scene graph relations, clinical role predictions and surgical phase recognition are assessed using precision, recall and F1-score, with a macroaverage computed over all relations, roles and phases, respectively. The macroaverage is sample size-agnostic, ensuring equal importance for all classes, which is essential in our setting since rare relation types such as *cutting* or *drilling* are crucial for accurate scene understanding. In all metrics, higher scores signify better performance.

## Results

**Fig. 5:**
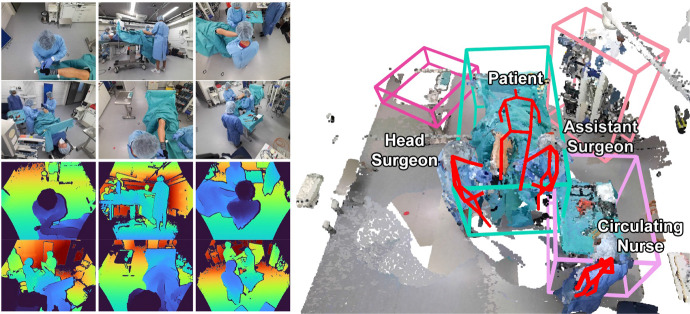
A sample 4D-OR scene with multiview RGB-D frames and fused 3D point cloud with detected 3D object bounding boxes, human poses and clinical roles

**Table 1:** Precision, recall and F1-scores for scene graph generation on test split, transposed

Relation	Assist	Cement	Clean	Close	Cut	Drill	Hammer	Hold	Lying	Operate	Prepare	Saw	Suture	Touch	None	Average
Prec	0.64	0.86	0.49	0.97	0.36	0.97	0.86	0.78	1.00	0.85	0.72	0.86	1.00	0.61	0.98	0.80
Rec	0.88	0.93	0.78	0.91	0.72	0.97	0.95	0.90	0.98	0.79	0.90	0.82	0.58	0.74	0.99	0.86
F1	0.74	0.89	0.60	0.94	0.48	0.97	0.90	0.84	0.99	0.82	0.80	0.84	0.73	0.67	0.98	0.81

“Average” stands for macroaverage, which is the unweighted average over all classes. We use images, augmentations, linear loss weighting and PointNet++

In this section, we present our results for human and object pose prediction, scene graph generation, clinical role prediction and surgical phase recognition.

**Human and object pose prediction:** We evaluate our method on the task of human pose recognition and achieve a PCP3D of 71.23 in the test split. For object pose recognition, our approach attains a high AP value of 0.9893 for IoU@25 and 0.9345 for IoU@50. The reliability of our methods is further corroborated by qualitative results visualized in Fig. 5, demonstrating accurate detection of human and object poses.

**Scene graph generation:** Our scene graph generation results, presented in Table 1, illustrate the effectiveness of relation prediction from a point cloud. We consider a relation “correct” if both entities are present in the scene and the relation between them is predicted accurately. Notably, our longer-trained method achieves the best result with a 0.81 macro-F1 using images and point clouds and the proposed augmentation strategies, which is 6% better than in our MICCAI paper. Figure 4 presents two qualitative scene graph generation examples, highlighting that our approach can successfully generate accurate scene graphs. However, our model occasionally fails in predicting the correct relation when instruments are occluded in scenes with high visual similarities but different tools (e.g., *drilling*, *sawing*).

**Table 2:** Precision, recall and F1-scores for clinical role prediction, comparing a heuristic-based method and a neural network-based Graphormer for track scoring

Role	Heuristic-based			Graphormer		
	Prec	Rec	F1	Prec	Rec	F1
Patient	0.99	0.98	0.99	0.99	0.92	0.96
Head surgeon	0.93	1.00	0.96	0.96	1.00	0.98
Assistant surgeon	0.74	0.74	0.74	0.87	0.96	0.91
Circulating nurse	0.65	0.60	0.62	0.91	0.86	0.88
Anesthetist	0.61	0.45	0.52	0.72	0.52	0.60
Macroaverage	0.78	0.75	0.77	**0**.**89**	**0**.**85**	**0**.**87**

Bold indicates the best result between heuristic based and graphormer based

**Clinical role prediction:** Table 2 presents our results for clinical role prediction. Near-perfect performance is achieved for patient and head surgeon roles, with good performance for assistant surgeon and circulating nurse roles. The anesthetist role, often partially occluded, proves challenging to predict accurately.

One potential remedy would be the incorporation of an additional camera to adequately cover the anesthetist’s workspace. Although the heuristic-based score assignment method yields lower scores, it retains the advantage of transparency, and not needing task-specific labels. Conversely, if such labels are available, a Graphormer-based approach might be easier to adapt to new roles or surgeries, as it does not require tweaking heuristics and leads to better results.

**Table 3:** Precision, recall and F1-scores for surgical phase recognition

Task	Prec	Rec	F1
OR preparation	0.88	1.00	0.94
Patient roll-in	0.99	0.94	0.97
Patient preparation	0.96	0.96	0.96
Surgery 1: implant placement preparation	0.95	0.95	0.95
Surgery 2: implant placement	0.98	1.00	0.99
Surgery 3: conclusion	1.00	0.98	0.99
Patient roll-out	0.96	0.98	0.97
OR cleanup	0.98	0.96	0.97
Macroaverage	0.96	0.97	0.97

**Surgical phase recognition:** Table 3 shows our results on surgical phase recognition. Our exceptional results show that our semantic scene graphs encode the information necessary to extract surgical phases. Furthermore, we achieve these results without relying on any surgical phase annotations, demonstrating the capability of scene graphs. The remainder of the errors is mainly caused by the ambiguity in phase transitions, where it is not always clear when one phase ends and the next one begins. While the findings from our simulated 4D-OR dataset suggest promising surgical phase recognition capabilities, it is imperative to validate these results in a real OR setting.

**Ablation studies:** We conduct ablation studies Table 4 to assess the impact of our contributions, including the use of images and augmentations. We also investigate the effects of employing ground truth human and object pose annotations instead of predictions. Our results demonstrate that using images (c-d) and augmentations (a-c) significantly improves F1 results, performing optimally when both are applied (a-d), thus validating the benefits of our method. Moreover, using ground truth instead of predictions (d-e) results in minimal change, indicating that our method can effectively utilize off-the-shelf pose prediction techniques. We further notice that our final model can be trained longer, which leads to even higher results (d-f).

**Table 4:** SSG generation using 3D point clouds with different configurations

Exp #	Image	Augment	GT	Longer training	F1
(a)	$\times$	$\times$	$\times$	$\times$	0.65
(b)	$\checkmark$	$\times$	$\times$	$\times$	0.66
(c)	$\times$	$\checkmark$	$\times$	$\times$	0.70
(d)	$\checkmark$	$\checkmark$	$\times$	$\times$	0.76
(e)	$\checkmark$	$\checkmark$	$\checkmark$	$\times$	0.78
(f)	$\checkmark$	$\checkmark$	$\times$	$\checkmark$	0.81

## Discussion and conclusion

In summary, our work contributes substantially to the field of holistic OR modeling by introducing the innovative concept of semantic scene graphs. We developed 4D-OR dataset, the first open-source dataset in the 4D-OR domain. Through our multimodal neural network-based pipeline, we generate semantic scene graphs, which offer valuable insights and decision-making support during surgical procedures. Our pipeline’s utility is demonstrated in critical tasks such as clinical role prediction and surgical phase recognition, signifying a meaningful stride toward the advancement of computer-assisted interventions. While this paper establishes a pathway for comprehensive modeling of surgical procedures, several challenges must be addressed before these methods can be fully implemented in clinical practice. Significant hurdles include data privacy concerns and the complexities associated with acquiring, storing and utilizing hospital data. Nevertheless, the potential benefits of our proposed approach, along with the broader advantages of Surgical Data Science solutions, will drive the research community to develop effective strategies to overcome these limitations.

Looking ahead, we envision expanding our solution to incorporate a broader range of modalities. This expansion could encompass integrating laparoscopic camera feeds, medical images, data from tools and digital equipment, audio signals and patient-specific electronic health records. By incorporating these additional inputs, we can create a more comprehensive multimodal semantic scene graph, leading to a more detailed and robust representation of the OR [31]. While our initial study does not yet confirm the clinical use of SSG in real surgeries, the holistic understanding of the simulated cases in the 4D-OR dataset validates the concept and shows the potential for applications in the clinical setting. Our modeling holds immense potential to benefit the research community, facilitating the conceptualization and realization of the future digital OR. For instance, it can enable the prediction of the impact of new technologies on overall workflow and facilitate the adaptation of digital equipment functions to the dynamic OR environment and their interaction with each other, ultimately resulting in enhanced patient outcomes. This promising direction not only underscores the significance of our current work but also paves the way for future advancements in this exciting field.

## Data Availability

Data, code and/or material is available at github.com/egeozsoy/4D-OR.

## Footnotes

1 https://azure.microsoft.com/en-us/services/kinect-dk/.
